# Supplementary material for: Identification and Bioinformatic Analysis of the GmDOG1-Like Family in Soybean and Investigation of Their Expression in Response to Gibberellic Acid and Abscisic Acid
Source: Plants (Basel). 2020 Jul 24;9(8):937. doi: 10.3390/plants9080937 (PMC7465105; doi:10.3390/plants9080937)
Supplement: Supplementary file 1 [file plants-09-00937-s001.zip › Supplementary-Table 4.docx]

**Table S4. Primers sequence used in this study.**

| **Gene name** | **Forward primer** | **Reverse primer** |
| --- | --- | --- |
| *GmTubulin* | AACCTCCTCCTCATCGTACT | GACAGCATCAGCCATGTTCA |
| *GmDOG1-L1* | GCCTTTGGGGAATGGTCAAG | CAGTCTACGAAGGGTTGGGA |
| *GmDOG1-L2* | CTCCAAGGATGCGACAAAGAC | GCTTGACTCGGCTGCTCTC |
| *GmDOG1-L3* | AGTGTTCAAGACCAAAAACGAATG | CGTGCATAGGCTGAGATGGG |
| *GmDOG1-L10* | CGGACTCTGTAGGACCAGGA | ACCTTGAAAAGAGACAACTTCCTC |
| *GmDOG1-L11* | CAAGGCCAAGTCAAGGTPTG | GGTCTCCATCCACCAATCCAA |
| *GmDOG1-L26* | GTGTGTCGATTTCTTGGCTGC | GATCCTGCAGCAGCAATGTCC |
| *GmDOG1-L27* | TAGCAACGGACGGAAGAGAGC | GGGACTGAAGCTCCTCGAAC |
| *GmDOG1-L30* | CTGGTCAAGCTCACCACTCG | CGCAAGAACCCCTTTCAACG |
| *GmDOG1-L37* | CCTCCTCCTCATCATCAGTTG | TCCGGAATCCTTGCTTAGGTAG |
| *GmDOG1-L39* | CAGTTAAACCAGAGCAGCAGG | CTGAAGGTGTACCCCTGCACA |
