# Supplementary material for: Identification and Bioinformatic Analysis of the GmDOG1-Like Family in Soybean and Investigation of Their Expression in Response to Gibberellic Acid and Abscisic Acid
Source: Plants (Basel). 2020 Jul 24;9(8):937. doi: 10.3390/plants9080937 (PMC7465105; doi:10.3390/plants9080937)
Supplement: Supplementary file 1 [file plants-09-00937-s001.zip › Supplementary-Table 3.docx]

**Table S3.** **Duplication events of *GmDOG1Ls.***

| Segment pairs | KA | KS | KA/KS |
| --- | --- | --- | --- |
| *GmDOG1-L2 & GmDOG1-L1* | 1.0054 | 0.980744 | 1.02514 |
| *GmDOG1-L10 & GmDOG1-L15* | 0.026822 | 0.127302 | 0.210693 |
| *GmDOG1-L10 & GmDOG1-L21* | 0.165182 | 0.671999 | 0.245806 |
| *GmDOG1-L10 & GmDOG1-L34* | 0.155459 | 0.606815 | 0.256189 |
| *GmDOG1-L14 & GmDOG1-L35* | 0.032175 | 0.174753 | 0.184114 |
| *GmDOG1-L15 & GmDOG1-L34* | 0.146342 | 0.636186 | 0.23003 |
| *GmDOG1-L16 & GmDOG1-L3* | 1.01075 | 0.964682 | 1.04775 |
| *GmDOG1-L17 & GmDOG1-L39* | 0.063202 | 0.0949659 | 0.665523 |
| *GmDOG1-L18 & GmDOG1-L38* | 0.036007 | 0.150211 | 0.239711 |
| *GmDOG1-L21 & GmDOG1-L15* | 0.160585 | 0.743006 | 0.216129 |
| *GmDOG1-L21 & GmDOG1-L34* | 0.0156 | 0.098186 | 0.158885 |
| *GmDOG1-L24 & GmDOG1-L29* | 0.016661 | 0.0679639 | 0.245148 |
| *GmDOG1-L28 & GmDOG1-L23* | 0.017854 | 0.0870612 | 0.205077 |
| *GmDOG1-L32 & GmDOG1-L25* | 1.00707 | 0.976353 | 1.03146 |
| *GmDOG1-L36 & GmDOG1-L4* | 0.015759 | 0.0842581 | 0.187031 |
| *GmDOG1-L37 & GmDOG1-L6* | 0.01719 | 0.127547 | 0.134774 |
