# Supplementary material for: Identification and Bioinformatic Analysis of the GmDOG1-Like Family in Soybean and Investigation of Their Expression in Response to Gibberellic Acid and Abscisic Acid
Source: Plants (Basel). 2020 Jul 24;9(8):937. doi: 10.3390/plants9080937 (PMC7465105; doi:10.3390/plants9080937)
Supplement: Supplementary file 1 [file plants-09-00937-s001.zip › Supplementary-Table 2.docx]

**Table S2. Information about the duplicated regions of *GmDOGL1s.***

| **Gene name start end chromosome** | |  |  | |  |
| --- | --- | --- | --- | --- | --- |
| \| *GmDOG1-L1* \| 24682089 \| 24688981 \| 1 \| \| --- \| --- \| --- \| --- \| \| *GmDOG1-L2* \| 8834516 \| 8841154 \| 2 \| \| *GmDOG1-L3* \| 29019977 \| 29028838 \| 2 \| \| *GmDOG1-L4* \| 34171531 \| 34177989 \| 3 \| \| *GmDOG1-L6* \| 35809857 \| 35816983 \| 3 \| \| *GmDOG1-L10* \| 37029299 \| 37037313 \| 5 \| \| *GmDOG1-L14* \| 18394479 \| 18395180 \| 7 \| \| *GmDOG1-L15* \| 10717490 \| 10725490 \| 8 \| \| *GmDOG1-L16*  *GmDOG1-L17* \| 12738719  42686749 \| 12747206  42691742 \| 10  10 \| \| *GmDOG1-L18* \| 49858890 \| 49863059 \| 10 \| \| *GmDOG1-L21* \| 33112560 \| 33118486 \| 11 \| \| *GmDOG1-L23* \| 34579255 \| 34588903 \| 12 \| \| *GmDOG1-L24* \| 19659235 \| 19664811 \| 13 \| \| *GmDOG1-L25* \| 30682181 \| 30695761 \| 13 \| \| *GmDOG1-L28* \| 41139769 \| 41147330 \| 13 \| \| *GmDOG1-L29* \| 41307402 \| 41312938 \| 14 \| \| *GmDOG1-L32* \| 43630502 \| 43639894 \| 15 \| \| *GmDOG1-L34* \| 1529592 \| 1536646 \| 18 \| \| *GmDOG1-L35* \| 48401099 \| 48401797 \| 18 \| \| *GmDOG1-L36* \| 38988741 \| 38995145 \| 19 \| \| *GmDOG1-L37* \| 40603618 \| 40611089 \| 19 \| \| *GmDOG1-L38* \| 35556507 \| 35561209 \| 20 \| \| *GmDOG1-L39* \| 43349627 \| 43352706 \| 20 \| |  | |  | |  |
|  | | | |  |  |
