# Supplementary material for: Identification and Bioinformatic Analysis of the GmDOG1-Like Family in Soybean and Investigation of Their Expression in Response to Gibberellic Acid and Abscisic Acid
Source: Plants (Basel). 2020 Jul 24;9(8):937. doi: 10.3390/plants9080937 (PMC7465105; doi:10.3390/plants9080937)
Supplement: Supplementary file 1 [file plants-09-00937-s001.zip › Supplementary-Table 1.docx]

**Table S1. Gene Name and Gene ID of *DOG1-Like* in 4 species**

| **Gene ID** | **Gene Name** |
| --- | --- |
| *AT5G45830.1* | *AtDOG1* |
| *AT4G18660.1* | *AtDOG1-L1* |
| *AT4G18680.1* | *AtDOG1-L2* |
| *AT4G18690.1* | *AtDOG1-L3* |
| *AT4G18650.1* | *AtDOG1-L4* |
| *AT1G08320.1* | *AtDOG1-L5* |
| *AT1G09950.1* | *AtDOG1-L6* |
| *AT1G15320.1* | *AtDOG1-L7* |
| *AT1G22070.1* | *AtDOG1-L8* |
| *AT1G58330.1* | *AtDOG1-L9* |
| *AT1G68640.1* | *AtDOG1-L10* |
| *AT1G77920.1* | *AtDOG1-L11* |
| *AT3G12250.1* | *AtDOG1-L12* |
| *AT3G14880.1* | *AtDOG1-L13* |
| *AT4G22600.1* | *AtDOG1-L14* |
| *AT5G06839.1* | *AtDOG1-L15* |
| *AT5G06950.1* | *AtDOG1-L16* |
| *AT5G06960.1* | *AtDOG1-L17* |
| *AT5G10030.1* | *AtDOG1-L18* |
| *AT5G65210.1* | *AtDOG1-L19* |
| *LOC_Os01g06560.1* | *OsDOG1-L1* |
| *LOC_Os01g17260.1* | *OsDOG1-L2* |
| *LOC_Os01g20030.1* | *OsDOG1-L3* |
| *LOC_Os01g59350.1* | *OsDOG1-L4* |
| *LOC_Os01g64020.1* | *OsDOG1-L5* |
| *LOC_Os02g10140.1* | *OsDOG1-L6* |
| *LOC_Os02g44250.1* | *OsDOG1-L7* |
| *LOC_Os03g20310.1* | *OsDOG1-L8* |
| *LOC_Os04g54474.1* | *OsDOG1-L9* |
| *LOC_Os05g37170.1* | *OsDOG1-L10* |
| *LOC_Os05g41280.1* | *OsDOG1-L11* |
| *LOC_Os05g48650.1* | *OsDOG1-L12* |
| *LOC_Os06g15480.1* | *OsDOG1-L13* |
| *LOC_Os06g41100.1* | *OsDOG1-L14* |
| *LOC_Os07g48820.1* | *OsDOG1-L15* |
| *LOC_Os08g07970.1* | *OsDOG1-L16* |
| *LOC_Os09g10840.1* | *OsDOG1-L17* |
| *LOC_Os09g31390.1* | *OsDOG1-L18* |
| *LOC_Os11g05480.1* | *OsDOG1-L19* |
| *LOC_Os12g05680.1* | *OsDOG1-L20* |
| *TraesCS3B02G120900.1* | *TaDOG1-L1* |
| *TraesCS1A02G096300.1* | *TaDOG1-L2* |
| *TraesCS1A02G276600.1* | *TaDOG1-L3* |
| *TraesCS3A02G306200.1* | *TaDOG1-L4* |
| *TraesCS1A02G392200.1* | *TaDOG1-L5* |
| *TraesCS1B02G127400.1* | *TaDOG1-L6* |
| *TraesCS1B02G285800.1* | *TaDOG1-L7* |
| *TraesCS1B02G420300.1* | *TaDOG1-L8* |
| *TraesCS1D02G105300.1* | *TaDOG1-L9* |
| *TraesCS1D02G276100.1* | *TaDOG1-L10* |
| *TraesCS1D02G400300.1* | *TaDOG1-L11* |
| *TraesCS2A02G010800.1* | *TaDOG1-L12* |
| *TraesCS2A02G097500.1* | *TaDOG1-L13* |
| *TraesCS2A02G486100.1* | *TaDOG1-L14* |
| *TraesCS2A02G526300.1* | *TaDOG1-L15* |
| *TraesCS2B02G113200.1* | *TaDOG1-L16* |
| *TraesCS2B02G512700.1* | *TaDOG1-L17* |
| *TraesCS2B02G556600.1* | *TaDOG1-L18* |
| *TraesCS2D02G096800.1* | *TaDOG1-L19* |
| *TraesCS2D02G529000.1* | *TaDOG1-L20* |
| *TraesCS3A02G103500.1* | *TaDOG1-L21* |
| *TraesCS3A02G190700.1* | *TaDOG1-L22* |
| *TraesCS3A02G334100.1* | *TaDOG1-L23* |
| *TraesCS3A02G372400.1* | *TaDOG1-L24* |
| *TraesCS3B02G220400.1* | *TaDOG1-L25* |
| *TraesCS3B02G330400.1* | *TaDOG1-L26* |
| *TraesCS3B02G365100.1* | *TaDOG1-L27* |
| *TraesCS3B02G404800.1* | *TaDOG1-L28* |
| *TraesCS3D02G105800.1* | *TaDOG1-L29* |
| *TraesCS3D02G194800.1* | *TaDOG1-L30* |
| *TraesCS3D02G295800.1* | *TaDOG1-L31* |
| *TraesCS3D02G327600.1* | *TaDOG1-L32* |
| *TraesCS3D02G365200.1* | *TaDOG1-L33* |
| *TraesCS4A02G126300.1* | *TaDOG1-L34* |
| *TraesCS4A02G183400.1* | *TaDOG1-L35* |
| *TraesCS4B02G135000.1* | *TaDOG1-L36* |
| *TraesCS4B02G178600.1* | *TaDOG1-L37* |
| *TraesCS4D02G129900.1* | *TaDOG1-L38* |
| *TraesCS4D02G180200.1* | *TaDOG1-L39* |
| *TraesCS5A02G174200.1* | *TaDOG1-L40* |
| *TraesCS5A02G265600.1* | *TaDOG1-L41* |
| *TraesCS5B02G265300.1* | *TaDOG1-L42* |
| *TraesCS5D02G178800.1* | *TaDOG1-L43* |
| *TraesCS5D02G273500.1* | *TaDOG1-L44* |
| *TraesCS6A02G165800.1* | *TaDOG1-L45* |
| *TraesCS6A02G248300.1* | *TaDOG1-L46* |
| *TraesCS6B02G193200.1* | *TaDOG1-L47* |
| *TraesCS6B02G276400.1* | *TaDOG1-L48* |
| *TraesCS6D02G154400.1* | *TaDOG1-L49* |
| *TraesCS6D02G230400.1* | *TaDOG1-L50* |
| *TraesCS7A02G207100.1* | *TaDOG1-L51* |
| *TraesCS7B02G114300.1* | *TaDOG1-L52* |
| *TraesCS7D02G209800.1* | *TaDOG1-L53* |
| *BAJ05340.1* | *HvDOG1-L1* |
| *AK248238.1* | *HvDOG1-L2* |
